# Portal vein embolization with absolute ethanol to induce hypertrophy of the future liver remnant

**DOI:** 10.1186/s42155-022-00312-3

**Published:** 2022-07-23

**Authors:** Cositha Santhakumar, William Ormiston, John L McCall, Adam Bartlett, David Duncan, Andrew Holden

**Affiliations:** 1 New Zealand Liver Transplant Unit, Auckland City Hospital, Auckland, New Zealand; 2 Department of Interventional Radiology, Auckland City Hospital, Auckland, New Zealand; 3 Hepatopancreaticobiliary Unit, Department of General Surgery, Auckland City Hospital, Auckland, New Zealand

**Keywords:** Portal vein embolization, Hepatectomy, Ethanol, Liver failure, Hypertrophy

## Abstract

**Background:**

Preoperative portal vein embolization (PVE) is widely used prior to major liver resection to reduce the risk of post-hepatectomy liver failure (PHLF). We evaluated the efficacy and safety of PVE using absolute ethanol.

**Methods:**

Consecutive patients undergoing preoperative PVE between February 2003 and February 2020 at a high-volume tertiary institution were retrospectively reviewed. Hypertrophy of the future liver remnant (FLR) was determined by comparing volumetric data using semi-automated software on computed tomography or magnetic resonance imaging before and after PVE. Efficacy of absolute ethanol was evaluated by the percentage increase in the FLR volume and the ratio of the FLR to the total liver volume (TLV). Technical success and complications following PVE were evaluated. Feasibility of hepatectomy following PVE and the incidence of PHLF were determined.

**Results:**

Sixty-two patients underwent preoperative PVE using absolute ethanol. The technical success rate was 95.2%. Median time interval between PVE and follow-up imaging was 34 days (range 6–144 days). The mean increase in FLR volume and ratio of the FLR to TLV were 43.6 ± 34.4% and 12.3 ± 7.7% respectively. Major adverse events occurred in 3 cases (4.8%) and did not preclude consideration of surgery. Forty-two patients (67.8%) proceeded to surgery for intended hepatectomy of which 36 patients (58.1%) underwent liver resection. Major post-operative complications occurred in 4 patients (11.1%) and there were no cases of PHLF.

**Conclusion:**

Preoperative PVE with absolute ethanol is effective and safe in inducing hypertrophy of the FLR before partial hepatectomy to prevent PHLF.

## Background

Liver resection provides a curative option for primary and metastatic hepatobiliary malignancies. The safety of major liver resection is contingent upon the future liver remnant (FLR) that remains following surgery. Furthermore, the risk of developing post-hepatectomy liver failure (PHLF) is directly related to the size and quality of the FLR (Blüthner et al. 2019). Portal vein embolization (PVE) can be utilised preoperatively to increase the FLR. In PVE, embolization of the portal vein branches that supply the part of the liver to be removed causes atrophy in these segments and concomitant hypertrophy of the FLR. This procedure has extended the surgical candidacy for patients previously unable to undergo major liver resection and has been associated with a reduced incidence of PHLF (May et al. 2013).

Several embolic materials and methods have been used for PVE but randomised controlled trials evaluating the efficacy of these various techniques are lacking. Absolute ethanol and n-butyl-cyanoacrylate (NBCA) glue have been reported to produce greater FLR hypertrophy (Sugawara et al. 2019; Ali et al. 2021). The advantages of absolute ethanol as an embolic agent include its strong contact destructivity, limited systemic toxicity and ease-of-use. It is easy to prepare for injection and penetrates deeply into the target vasculature providing complete obstruction of the portal vein branches and compensatory hypertrophy of the remaining lobes (Ogasawara et al. 1996). The mean percentage increase in the FLR using absolute ethanol in the literature has ranged from 33.6 − 46.5% (Yamakado et al. 1997; Sakuhara et al. 2012; Sofue et al. 2014; Yamamoto et al. 2016; Alvarez et al. 2018). However, comparisons between these studies are limited by differences in underlying liver disease, embolization technique, and timing between PVE and volumetry data analysis and FLR assessment. Therefore, both mean and median values for volumetric data are reported in this study to facilitate comparison with previous studies.

The objectives of this study were to firstly evaluate the efficacy and safety of PVE using absolute ethanol prior partial hepatectomy, and secondly to evaluate the incidence of PHLF and major post-operative complications.

## Methods

### Patient selection

**Fig. 1:**
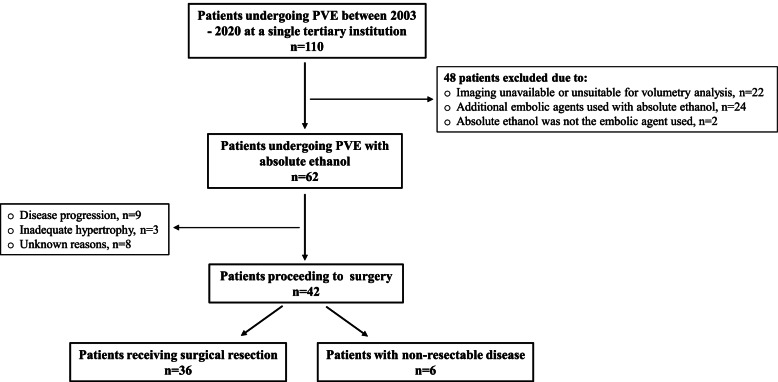
Flowchart of patients

Consecutive patients undergoing portal vein embolization prior to partial hepatectomy at a single tertiary institution between February 2003 and February 2020 were identified and reviewed. One hundred and ten patients were identified (Fig. 1). Forty-eight patients were excluded for the following reasons: imaging unavailable or unsuitable for volumetry analysis with the available software (*n* = 22), an additional embolic agent was used with absolute ethanol (*n* = 24), and absolute ethanol not used as an embolic material (*n* = 2). The final cohort consisted of 62 patients with a range of primary and secondary hepatobiliary lesions (Table 1).

All patients were referred for PVE from the Hepatobiliary unit or the National Liver Transplant Unit to the Department of Interventional Radiology after discussion at a multidisciplinary meeting where hepatic resectability was determined. The decision to undergo PVE was based on factors including the anticipated size of the FLR, underlying liver function, patient co-morbidity and complexity of the planned surgery. In the selected cases, PVE was performed to induce hypertrophy of the FLR prior to partial hepatectomy.

Relevant clinical and surgical information were retrieved from the hospital’s medical record database. The dominant liver parenchymal feature was categorised as follows: normal, fibrotic (including cirrhosis), steatotic or cholestatic, based on clinical or biochemical evaluation, imaging and histology (when available). Fibrosis was primarily determined from the histology of the resected specimen or transient elastography when available. Approval by the local ethics committee was not required to undertake this study as it is a review of existing clinical procedures and outcomes.

### Technique of PVE

Percutaneous transhepatic PVE was performed in 62 patients using absolute ethanol alone under general anaesthesia by experienced interventional radiologists at the same tertiary institution. No procedures were performed under sedation. The embolized portal branches were as follows: right portal vein in 57 patients (91.9%), right portal vein and Segment 4 branch in 2 patients (3.2%), and left portal vein in 3 patients (4.8%).

**Fig. 2:**
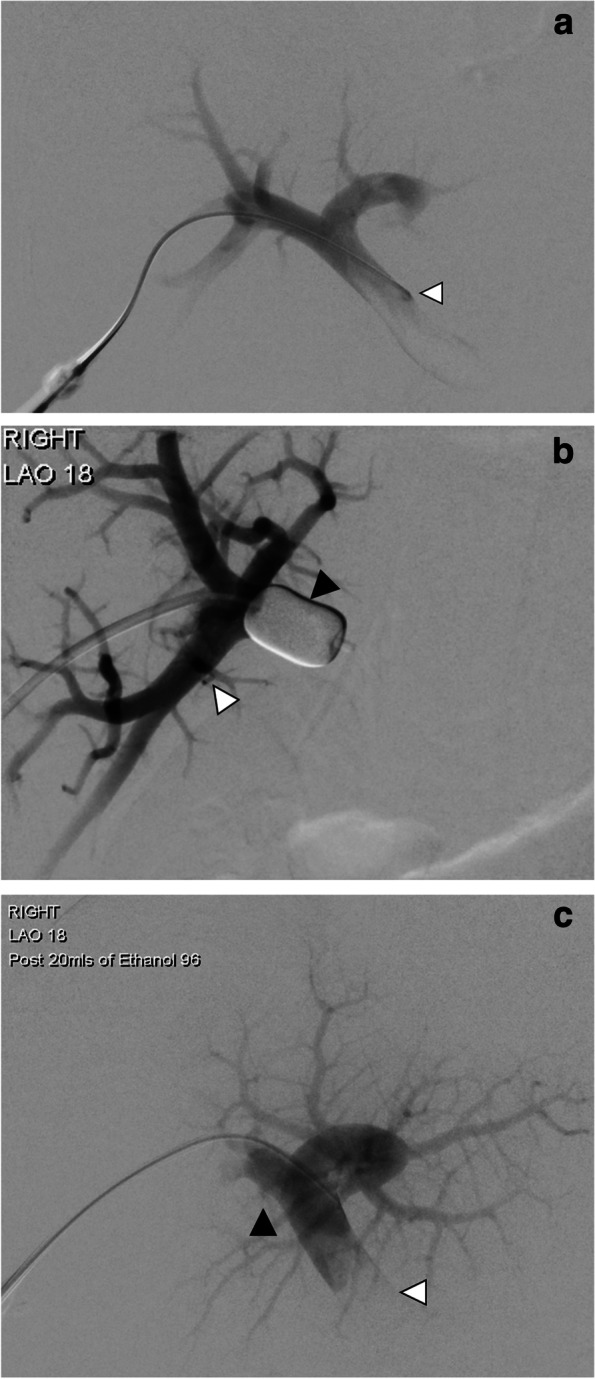
**a** Venography of the portal system via a transhepatic approach via catheter in the main portal vein (white arrow), which demonstrates portal anatomy prior to embolization. **b** Venography of the portal system via the sheath with the LeMaitre balloon inflated (black arrow), demonstrating adequate occlusion of the right portal system (white arrow). **c** Venography of the portal system, demonstrating patent main portal vein (white arrow) and successful occlusion of the right portal system (black arrow)

The procedural description is provided for right portal vein embolization. Under ultrasound guidance, percutaneous transhepatic access to a right portal vein branch is usually achieved and a 7 Fr sheath inserted over a guidewire. A catheter is inserted into the main portal vein (PV), and venography performed to confirm portal venous anatomy (Fig. 2a). A 5 or 6 Fr low pressure compliant balloon (Over-the-wire Embolectomy Catheter, Le Maitre Vascular Inc, Burlington MA, USA) is inflated in the proximal right PV. Repeat venography via the sheath whilst the compliant balloon is inflated is performed to confirm occlusion of the central right PV (Fig. 2b). The volume of contrast required to completely opacify the right portal venous system is recorded and used as guidance for subsequent 100% ethanol injection. The right portal venous system is then embolized by injecting 100% ethanol (typically 20–30ml) through the sheath and left to instil for 10 min. The occlusion balloon is then deflated. Completion venogram performed to confirm adequate thrombosis of the right portal venous system (Fig. 2c). If embolization was incomplete, further instillation of ethanol performed after reinflation of the compliant balloon in the appropriate position. In a minority of cases due to anatomic reasons, the right PV is embolized from a contralateral percutaneous left lobe approach. In this situation, the compliant balloon is positioned in the central right balloon, inflated and ethanol injected via the catheter lumen.

Adverse events relating to the PVE were classified according to the Society of Interventional Radiology Existing Adverse Event Classification (Khalilzadeh et al. 2017), according to local convention.

Follow-up imaging was performed following PVE using either contrast-enhanced computed tomography (CT) or magnetic resonance imaging (MRI). Volumetric assessment was subsequently performed (Section 2.3). Additional PVE procedures were undertaken if the FLR hypertrophy was deemed inadequate to facilitate the anticipated liver resection based on the post-embolization FLR and in the clinical context of the patient. In the event of interval disease progression precluding resection, alternative treatments were proposed.

### Volumetry

**Fig. 3:**
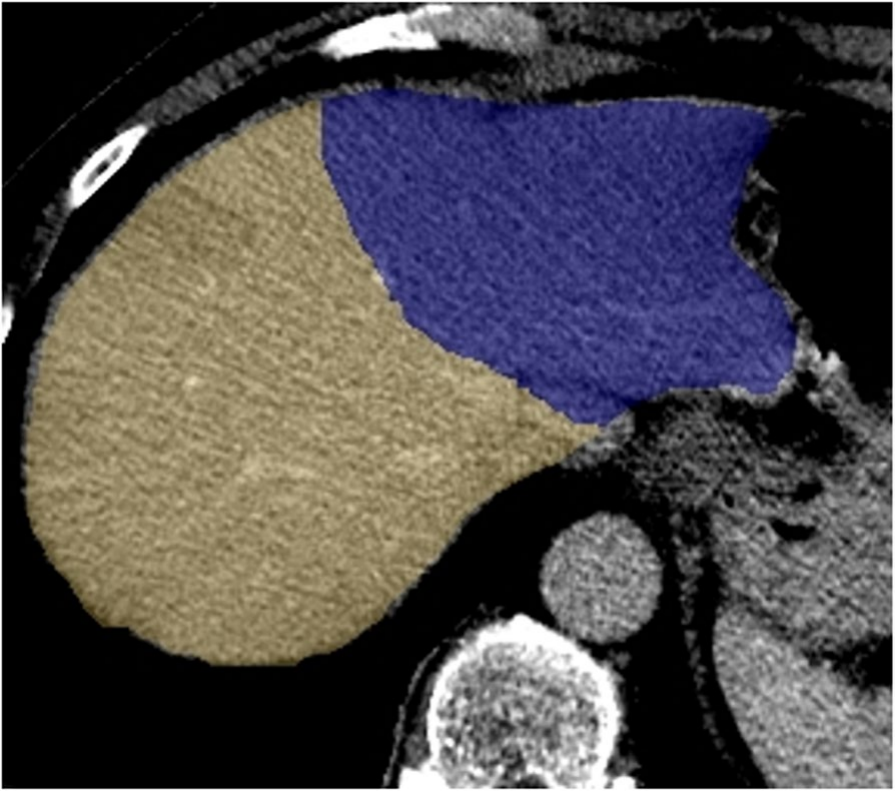
Semi-automated segmentation of contrast enhanced CT of the liver into left and right lobes

Liver volumes were measured on contrast-enhanced CT or MRI before and after PVE. Volumetry analysis was performed using semi-automated software (Philips IntelliSpace Portal) by a trained investigator (CS) with two years experience and confirmed by a Radiologist (WO) with seven years experience in volumetry (Fig. 3). CS was not blinded and WO was blinded. The area segmented was correlated with the areas embolized, for example if the right lobe was embolised, the FLR was the left lobe (segments 1, 2, 3 and 4). In 52 cases the same imaging modality (CT) was used for volumetric assessment before and after PVE, and in the remaining 10 cases, MRI was used before PVE and CT was used following PVE.

The change in the FLR (mL) and TLV (mL) before and after PVE was first determined. The degree of hypertrophy (DH%) was subsequently determined using two methods. Firstly, as the percentage change in the FLR volume: DH% = (FLR after PVE - FLR before PVE / FLR before PVE) x 100% and secondly, as a change in the FLR as a proportion of the TLV: DH% = (FLR/TLV after PVE – FLR/TLV before PVE) x 100%. The kinetic growth rate (KGR) was determined using the following formula: KGR (%) = DH at first post-PVE volume assessment (%) ÷ time elapsed since PVE (weeks) at first post-PVE volume assessment.

### Surgery

Surgical procedures were performed by experienced hepatobiliary surgeons at the same tertiary institution with over twenty-five years of experience. The decision to proceed to surgery following PVE was based on sufficient hypertrophy of the FLR on follow-up imaging to facilitate the anticipated surgery and the absence of disease progression that would preclude surgical resection. Major and minor hepatectomies were defined as the resection of ≥ 4 or < 4 Couinaud segments respectively.

Surgical outcomes within 30 days included major post-operative complications according to the Clavien-Dindo (Dindo et al. 2004) classification and PHLF according to the International Study Group of Liver Surgery (Rahbari et al. 2011) classification. Perioperative mortality was defined as mortality within 30 days following liver resection.

### Endpoints

The primary endpoint was the degree of hypertrophy determined by the percentage change in the FLR. Secondary endpoints were as follows: percentage increase in the FLR/TLV ratio after PVE, technical success and completeness of embolization, disease progression between PVE and follow-up imaging, complications following PVE, feasibility of hepatectomy after PVE, and incidence of post-operative complications and PHLF following liver resection. Technical success was defined as angiographic confirmation of portal venous occlusion performed by the Interventional Radiologist at the conclusion of the procedure. Completeness of embolization was defined as the absence of enhancement of the right portal vein on follow-up imaging. Disease progression between PVE and follow-up imaging was defined as radiological evidence of increased tumour burden at the follow-up scan.

### Statistics

Categorical data are presented as number of patients (%). Volumetric data are presented as mean ± SD (median, range). Non-parametric statistics were used throughout given the generally skewed nature of the data. The Mann-Whitney U test was used for comparison of two independent groups and the Kruskal-Wallis test for more than two groups. The Wilcoxon signed rank test was used for paired data.

SAS v.9.4 (SAS Institute, Cary, NC) was used for the statistical analyses. A p-value of less than 0.05 was considered statistically significant. Graphs were created using RStudio (Version 1.1.414).

## Results

A total of 62 patients met the inclusion criteria and were analysed. Baseline patient characteristics are presented in Table 1. The indication for liver resection was most commonly for hepatocellular carcinoma (37.1%), followed by colorectal liver metastases (CLRM, 32.3%), cholangiocarcinoma (21.0%), and other hepatobiliary lesions (9.7%). Twenty-one patients had underlying fibrosis (33.9%), of which 12 patients were cirrhotic, 8 patients (12.9%) had underlying steatosis and 12 patients (19.4%) had cholestasis. The median pre-interventional bilirubin was 10 µmol/L (3-310 µmol/L), albumin 38 g/L (23–47 g/L) and international normalised ratio 1.0 (0.9–1.3). Twenty-one (33.9%) patients had no underlying parenchymal liver disease. Forty-one patients underwent lesion other treatment/s prior to PVE: chemotherapy (*n* = 21), transarterial chemoembolization (TACE) (*n* = 5), ablation (*n* = 1), and biliary drainage via percutaneous transhepatic cholangiography or endoscopic retrograde cholangio-pancreatography (*n* = 14).

### Outcomes of PVE

**Fig. 4:**
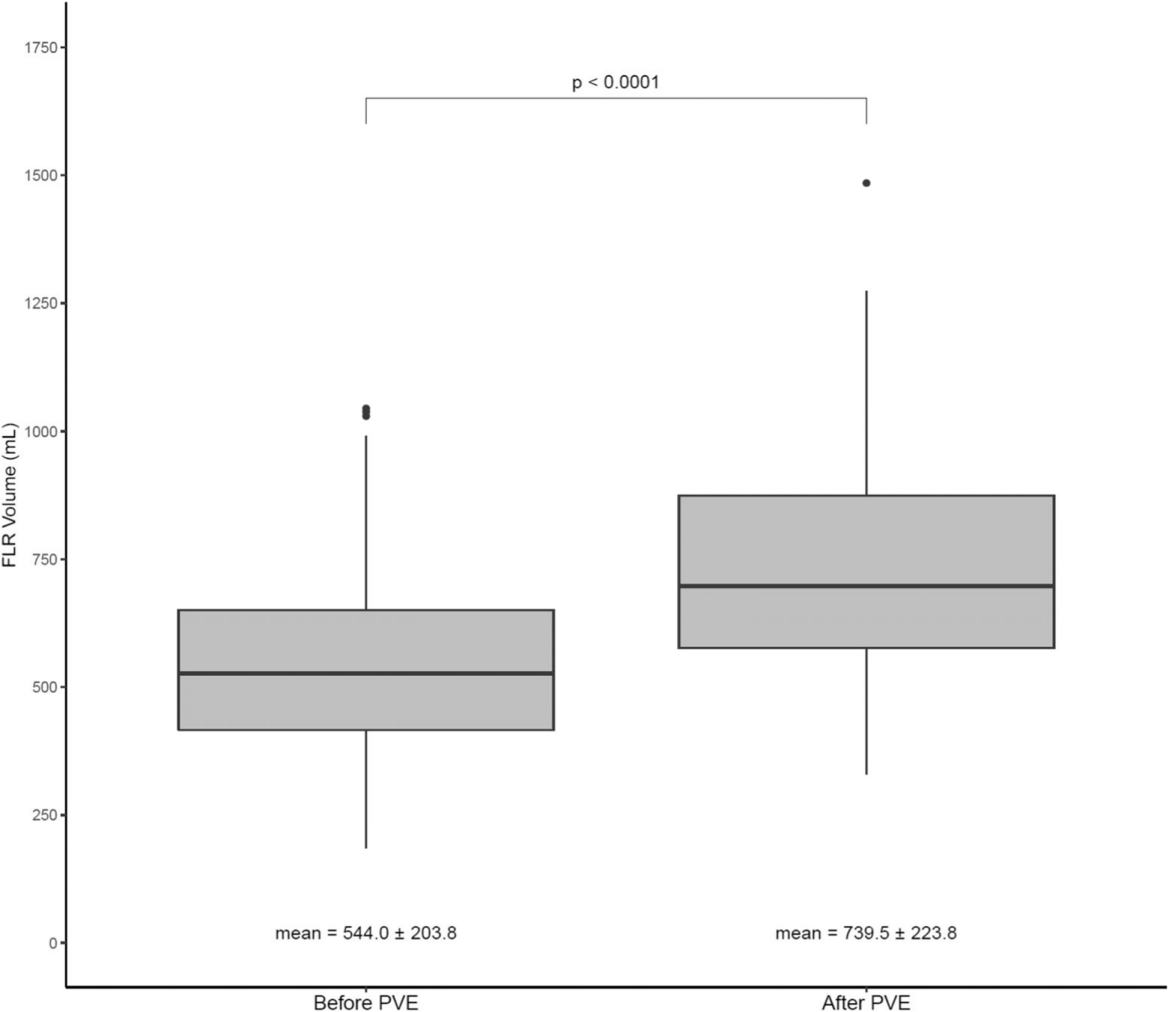
Mean FLR volume increased from 544.0 ± 203.8 mL before PVE to 739.5 ± 223.8 mL after PVE. This absolute increase in volume was statistically significant (*p* < 0.0001)

The median time interval from PVE to follow-up imaging was 34 days (range 6–144 days). The TLV changed from 1782.0 ± 516.5mL (1635.1 [828.6–3145.7] mL) before to 1771.1 ± 521.7 mL (1692.0 [746.5–3377.2] mL) after PVE (*p* = 0.378). The FLR volume significantly increased from 544.0 ± 203.8 mL (527.5 [185.0–1044.3] mL) before to 739.5 ± 223.8 mL (697.1 [330.0–1484.8] mL) after PVE (*p* < 0.0001) (Fig. 4). The degree of hypertrophy as a percentage increase in the FLR was 43.6 ± 34.4% (36.5 [-10.8–167.4]%). The change in the FLR as a proportion of the TLV was 12.3 ± 7.7% (10.0 [-3.2–39.0]%) (Table 2). The kinetic growth rate was 3.1% per week.

**Table 1:** Patient baseline characteristics

**Age (y), median (range)**	62.5 (9–80)
**Gender, n (%)**	
Male	47 (75.8)
Female	15 (24.2)
**Indication of Liver Resection, n (%)**	
Hepatocellular Carcinoma	23 (37.1)
Colorectal Metastases	20 (32.3)
Cholangiocarcinoma	13 (21.0)
Other	6 (9.7)
**Parenchymal Liver Disease, n (%)**	
Fibrosis	21 (33.9)
Steatosis	8 (12.9)
Cholestasis	12 (19.4)
None	21 (33.9)
**Pre-Interventional Bilirubin, median (µmol/L)**	10 (3-310)
**Pre-Interventional Albumin, median (g/L)**	38 (23–47)
**Pre-Interventional INR, median**	1.0 (0.9–1.3)
**Diabetes Mellitus, n (%)**	44 (71.0)

**Table 2:** Volumetric outcomes

**Pre-PVE Volumetry (ml), mean (SD)**	
Total Liver Volume	1782.0 (516.5)
Future Liver Remnant	544.0 (203.8)
**Post-PVE Volumetry (ml), mean (SD)**	
Total Liver Volume	1771.1 (521.7)
Future Liver Remnant	739.5 (223.8)
**Degree of Hypertrophy (%), mean (SD)**	
Change in Future Liver Remnant (%)	43.6 (34.4)
Change in Future Liver Remnant/Total Liver Volume Ratio (%)	12.3 (7.7)

According to aetiology, the degree of hypertrophy was 48.7% in CRLM, 42.5% in cholangiocarcinoma and 38.9% in HCC. There was no significant difference in the degree of hypertrophy between the aetiologies (*p* = 0.555) or according to the presence of underlying parenchymal disease (*p* = 0.197). The degree of hypertrophy was greatest in patients undergoing right and segment 4 embolization (53.6%), followed by right PVE (44.8%) and left PVE (12.7%).

PVE was technically successful in 59 patients (95.2%) at the time of the procedure and complete embolization occurred in 79.0%. Four patients (6.5%) required a repeat procedure due to incomplete embolization resulting in inadequate hypertrophy of the FLR. Disease progression between PVE and follow-up imaging was 41.9%.

Major adverse events occurred in 3 cases (4.8%) and minor adverse events occurred in 6 cases (9.7%). Major complications included iatrogenic right hepatic lobe injury (*n* = 1) and inadvertent right hepatic artery puncture (*n* = 2). Minor complications included pain requiring more than routine oral analgesia (*n* = 4), significant derangement in liver enzymes and international normalised ratio requiring intravenous Vitamin K (*n* = 1), and a mild allergic reaction that was managed with oral antihistamines (*n* = 1).

### Surgical Outcomes

Of the 62 patients, 42 patients (67.8%) proceeded to laparotomy for attempted liver resection. However, only 36 patients (58.1%) underwent liver resection of which 33 cases were major hepatectomies and 3 were minor hepatectomies. In the 25 patients who did not undergo resection, this was most commonly due to disease progression determined by progress imaging or unresectable disease at the time of laparotomy (*n* = 15, 24.2%) or inadequate hypertrophy to facilitate the anticipated surgery in three cases (4.8%). Of these three cases, one patient subsequently underwent TACE due to progression of HCC on follow-up imaging precluding resection. The remaining two cases had sufficient hypertrophy of the FLR following repeat embolization and proceeded to surgery but were abandoned at laparotomy due to the presence of unresectable disease in one case and the presence of portal hypertension in the other. Cholangiocarcinoma was the most common hepatobiliary lesion with disease progression precluding surgery (60%). In seven patients the reason for not proceeding to surgery was unknown and in one patient there was no available documentation to confirm if the patient proceeded to surgery following PVE. The median time to surgery following PVE was 56 days (range 17–218 days).

Post-operative complications occurred in 26 patients (72.2%), of which 4 (11.1%) were major and 22 (61.1%) were minor. There were no cases of PHLF and the post-operative mortality was 0%. Surgical outcomes are summarised in Table 3.

**Table 3:** Surgical outcomes

**Resection Post-PVE, n (%)**	
Yes	36 (58.1)
No	25 (40.3)
Disease Progression	15 (24.2)
Inadequate Hypertrophy	3 (4.8)
Unknown	7 (11.3)
Unknown	1 (1.6)
**Surgical Intervention**	
Major Hepatectomy	33 (93.9)
Minor Hepatectomy	3 (6.1)
**Timing of Surgery following PVE (days), median (range)**	56 (17–218)
**Post-Operative Complications within 30 days, n (%)**	
Yes	26 (72.2)
Major (≥ 3)	4 (11.1)
Minor (< 3)	22 (61.1)
No	10 (27.8)
**Post-Hepatectomy Liver Failure within 30 days, n (%)**	
Yes	0 (0.0%)
No	36 (100.0)

## Discussion

In this single-centre retrospective analysis, absolute ethanol was an effective, safe and feasible embolic material for PVE to induce hypertrophy of the FLR prior to partial hepatectomy. The mean degree of FLR hypertrophy using absolute ethanol in our study was 43.6% which is equivalent to other studies using absolute ethanol (Yamakado et al. 1997; Sakuhara et al. 2012; Sofue et al. 2014; Yamamoto et al. 2016; Alvarez et al. 2018) and other embolic agents (van Lienden et al. 2013). Various embolic materials are utilised for PVE and randomised prospective controlled trials comparing their efficacy are lacking. The most common embolic agents used include absolute ethanol, NBCA, poly-vinyl alcohol (PVA) particles, coils, vascular plugs, fibrin glue and gelatin foam (May et al. 2013). Our institution uses absolute ethanol because of its strong contact destructivity, ease-of-use, availability, short procedure time and low cost. Many of the alternative embolic agents require sub-selective catheterization of multiple portal vein segmental branches which can be technically challenging, time consuming and expensive. Furthermore, pre-clinical models using absolute ethanol for PVE have shown rapid and complete vascular obliteration of the portal vein with massive necrosis at the affected region and hepatic regeneration (Ogasawara et al. 1996). Absolute ethanol also permeates peripherally resulting in complete obstruction of peripheral portal branches whilst preserving bile ducts and large vessels in the embolized lobe and liver function in the remaining lobes (Ogasawara et al. 1996). A recent systematic review and meta-analysis compared five embolic materials and found NBCA to be superior in inducing growth of the FLR (Ali et al. 2021). However, the majority of included studies were small, single-centre and retrospective, highlighting the need for randomised studies comparing available embolic materials.

Complications relating to PVE at our institution occurred in 14.5% of cases and is in line with other reports (Kodama et al. 2002; Stefano et al. 2005). Major complications occurred in 4.8% of cases. Major complications were not thought to be secondary to the use of ethanol and did not preclude consideration of surgery. These results are comparable to the 11% and 6% thresholds for PVE-related morbidity and major complications respectively proposed by the Society of Interventional Radiologists quality improvement guidelines (Angle et al. 2010). Due to the retrospective nature of this study, we are unable to determine if the complications following PVE were directly related to the injection of ethanol.

The minimal requirement of FLR in patients with a normal liver is between 20 and 30% and for injured livers (chemotherapy, chronic hepatitis/cirrhosis) lies between 30 and 50% using CT based non-tumour volume or formula-based volume (Kawaguchi et al. 2019). At our institution, the clinical decision to proceed with PVE is based on the anticipated volume of the FLR postoperatively together with the baseline liver function, presence of underlying liver disease, patient factors and complexity of the planned surgery, rather than a specific cut-off for the FLR. This decision is made in a multidisciplinary setting involving hepatobiliary surgeons, hepatologists, radiologists and interventional radiologists.

Forty-two patients (67.8%) patients proceeded to surgery for intended liver resection but only 36 patients (58.1%) underwent surgery. The resection rate in this study is lower than other studies (Sakuhara et al. 2012; Sofue et al. 2014; Yamamoto et al. 2016; Alvarez et al. 2018) and was most commonly due to disease progression precluding resection (24.2%) rather than inadequate hypertrophy of the FLR (3.8%). Perhaps the longer time interval between PVE and the follow-up imaging in this study compared with other studies may account for this finding. Similar to the findings by Alvarez et al. (Alvarez et al. 2018), tumours arising from a biliary origin represented the highest proportion of cases with disease progression precluding curative resection.

More recently, other techniques have been proposed to hypertrophy the FLR including associating liver partition and portal vein ligation (ALPPS), transhepatic liver venous deprivation (LVD) and radiation lobectomy with radioactive yttrium 90 microspheres. ALPPS involves transecting the liver at the time of portal vein ligation during the first stage of a two-stage hepatectomy (Schnitzbauer et al. 2012). When compared with PVE, despite a greater increase in the FLR volume, ALPPS has demonstrated trends towards a higher morbidity (73% vs. 59%), mortality (14% vs. 7%) (Eshmuminov et al. 2016) and incidence of PHLF ranging from 8.3 to 14% (Schadde et al. 2015; Olthof et al. 2017). Subsequently, LVD, a technique where simultaneous embolization of the portal and hepatic veins was found to induce greater hypertrophy of the FLR than PVE alone without an increase in mortality or morbidity in cohort studies (Heil and Schadde 2021). Radiation lobectomy with yttrium 90 can also be performed to induce hypertrophy of the FLR. It has the concomitant advantage of controlling the liver tumour and limiting tumour progression in the contralateral untreated lobe by limiting the rate of portal blood flow (Vouche et al. 2013). All cases in our study were performed under general anaesthesia, primarily for pain control, however alternative techniques, particularly radiation lobectomy, can be performed under local anaesthesia or sedation.

Our study has certain limitations. Firstly, disease progression between PVE and follow-up imaging in our study was higher (41.9%) than other studies (Pandanaboyana et al. 2015) although direct comparisons between studies are flawed due to different time intervals between embolization and follow-up imaging. Accelerated tumour growth has been reported for both primary and secondary liver tumours (Kokudo et al. 2001; Hayashi et al. 2007) following PVE and chemotherapy has not been shown to prevent disease progression between PVE and liver resection (May et al. 2013). Disease progression was defined by an increase in tumour burden and not according to the RECIST criteria which represents a further limitation of our study. Secondly, in 10 cases, a different modality (either CT or MRI) was used before and after the embolization. Excluding these cases, the degree of FLR hypertrophy was similar (47.1 ± 35.5%, [40.1 {-10.8–167.4}%]). Finally, the retrospective and non-randomized design of the current study limited further analysis and introduces a selection bias.

## Conclusions

In conclusion, PVE using absolute ethanol is simple, effective and safe in inducing hypertrophy of the FLR to prevent PHLF after partial hepatectomy.

## Data Availability

The datasets used and/or analysed during the current study are available from the corresponding author on reasonable request.

## Abbreviations

PVE: Portal vein embolization
PHLF: Post-hepatectomy liver failure
FLR: Future liver remnant
TLV: Total liver volume
NBCA: N-butyl-cyanoacrylate
PV: Portal vein
CT: Computed tomography
MRI: Magnetic resonance imaging
KGR: Kinetic growth rateCLRM: colorectal liver metastases
INR: International normalised ratio
TACE: Transarterial chemoembolization
PVA: Poly-vinyl alcohol
ALPPS: Associating liver partition and portal vein ligation
LVD: Liver venous deprivation
